# Effect of annealing using plasma-activated water on the structure and properties of wheat flour

**DOI:** 10.3389/fnut.2022.951588

**Published:** 2022-08-11

**Authors:** Yizhe Yan, Xinhuan Xue, Xueyuan Jin, Bin Niu, Zhenzhen Chen, Xiaolong Ji, Miaomiao Shi, Yuan He

**Affiliations:** ^1^College of Food and Bioengineering, Henan Key Laboratory of Cold Chain Food Quality and Safety Control, Zhengzhou University of Light Industry, Zhengzhou, China; ^2^School of Clinical Medicine, Hainan Vocational University of Science and Technology, Haikou, China; ^3^College of Food Science and Technology, Henan Agricultural University, Zhengzhou, China

**Keywords:** plasma-activated water, annealing, wheat flour, structure, properties

## Abstract

In this study, wheat flour (WF) was modified by annealing (ANN) using plasma-activated water (PAW) for the first time. Compared with WF and DW-WF, the results of scanning electron microscopy (SEM) and particle-size analysis showed that the granule structure of wheat starch in PAW-WF was slightly damaged, and the particle size of PAW-WF was significantly reduced. The results of X-ray diffraction and Fourier transforming infrared spectroscopy indicated that PAW-ANN could reduce the long-range and short-range order degrees of wheat starch and change the secondary structure of the protein in WF, in which the content of random coils and α-helices was significantly increased. In addition, the analysis of solubility, viscosity, and dynamic rheological properties showed that PAW-ANN improved the solubility and gel properties of WF and decreased its viscosity properties and short-term regeneration. PAW-ANN, as a green modification technology, has the potential for further application in WF modification, as well as in the production of flour products.

## Introduction

Wheat is a cereal crop that is widely grown all over the world. After being ground into wheat flour (WF), it is widely used in food and can be used to make bread, steamed bread, biscuits, noodles, and other foods (1). The quality of flour products is often related to the functional properties of WF, such as gelatinization and gelling properties. However, due to the limitations of milling process and equipment, it is difficult for WF to meet the requirements of a specific food. Therefore, the modification of WF is required to expand its application. In addition to modification by chemical and enzymatic methods, physical methods have also become increasingly important due to good safety. The most used methods of physical modification include hydrothermal, extrusion, microwave, irradiation, and micronization. Bhat et al. irradiated WF and found that the water absorption, oil absorption, and swelling power (SP) of WF decreased, while its water solubility index, stability, and foaming ability increased (2). Lazaridou et al. showed that the reduction in the particle size of WF by micronization techniques largely affected their functional properties, such as water absorption and the amount of damaged starch, as well as the extractability of their components, such as arabinoxylan and molecular properties, which are important factors in determining starch gelatinization and dough rheological properties in WF (3).

Annealing (ANN) is a physical modification method that is usually carried out under excessive water (>40%) and low temperature above the glass transition temperature but below the gelatinization temperature for a period (usually more than 16 h). ANN conditions are relatively mild, involving only water and heat, so it has attracted wide attention (4). The structural and functional properties of starch and starch-based products are changed after ANN, making them easier to process and use in some unique environments.

The water obtained by the plasma discharge treatment of distilled water is called plasma-activated water (PAW). Plasma equipment can make the water environment acidic, increase redox potential, and enhance electrical conductivity by the generation of reactive oxygen species (ROS) and reactive nitrogen species (RON). PAW is gentler than direct plasma treatment. It uses water as the medium to avoid direct plasma damage to the surface caused by specific substances such as charged particles, ultraviolet rays, and electrons. Meanwhile, it exists in the form of liquid, which is more convenient and flexible. It can directly use air as a working gas and distilled water as an activation liquid, with low cost and no secondary pollution. Distinctive physicochemical properties make it widely employed in the food industry (5). Recently, PAW has been used to modify the structure and improve the properties of the starch by our group (6, 7). The multi-scale structure of various starches was modified, and their physicochemical and digestive properties were also improved by PAW combined with heat-moisture treatment or ANN. Wheat starch, as the main component of WF, is related to the properties of WF. Therefore, PAW modification could provide a possible way to change the structure and properties of WF.

In this study, PAW and ANN were used to synergistically modify WF, changing the multi-scale structure of starch and protein in WF, and improving the physicochemical properties of WF. The dual physical modification method is simple, convenient, green, and safe, avoiding the use of chemical substances or biological enzymes, and has the potential for modification of flour in the future.

## Materials and methods

### Materials

WF was obtained from COFCO Grain and Oil Co., Ltd. (Zhengzhou, China). All chemicals used were of analytical grade.

### Preparation and characterization of plasma-activated water

In this study, PAW was prepared by an atmospheric pressure plasma jet device (Easton Geake Automation Equipment Co., Ltd., Shenzhen, China). Approximately 100 ml of distilled water (DW) was placed in a cylindrical plastic bottle with the plasma nozzle 2 cm from the water surface. PAW was obtained after plasma jet treatment for 2 min under high frequency and high pressure (40 kHz, 5 kV). The pH value, oxidation-reduction potential (ORP), and conductivity of PAW were determined by the pH/ORP meter and conductivity meter (Yidian Scientific Instrument Co., Ltd., Shanghai, China). Notably, PAW was suggested to be further used within 12 h.

### Annealing treatment of wheat flour with distilled water or plasma-activated water

WF (25 g, dry basis) was placed in a Petri dish and dried at 50°C until about 5% moisture content. DW and PAW were used to adjust moisture content to 70%, respectively. After stirring and mixing, WF was put into hydrothermal reactors and then placed into an oven at 50°C for 12 h. After cooling to room temperature, WF was poured into a Petri dish, lyophilized, ground, and sieved to obtain the modified WF. These modified WF samples were denoted as DW-WF and PAW-WF. All samples are prepared in triplicates for further analysis.

### Scanning electron microscopy

The samples were evenly attached to the sample stage with conductive adhesive, sprayed with gold for 120 s in the ion sputtering device, and then observed under a high-resolution field emission scanning electron microscope (Regulus 8100, Hitachi, Tokyo, Japan). Under the accelerating voltage of 3 kV, the surface microstructure of the samples was observed at 1,000 times magnification.

### Particle size analysis

The samples to be tested were prepared into a flour emulsion (1%, w/w). The samples were tested for particle size distribution using a laser particle size analyzer (LS13320/ULM2, Beckman Coulter Ltd., United Kingdom). The corresponding data were recorded and analyzed.

### X-ray diffraction

Before the test, the samples were placed in saturated NaCl solution for 1 week at room temperature to equilibrate the moisture (8). An appropriate amount of WF sample powder was placed on the circular test board and compacted with a smooth glass sheet. The samples were analyzed using an X-ray diffractometer (D8 Advance, Bruker, Karlsruhe, Germany). The test conditions are as follows: 40 kV pipe pressure, 30 mA pipe flow, 4°/min scan speed, 5°–35° scanning area, 0.02° step, continuous scanning, and repeated once. The relative crystallinity (RC) of flour samples was calculated according to our previous method (6).

### Fourier transform infrared spectroscopy

Before the test, the potassium bromide required for the test was dried at 105°C for 5 h. WF sample and potassium bromide, according to the mass ratio of 1:100, were mixed, ground, and pressed into the tablet. A Fourier transform infrared (FTIR) spectrometer (Vertex 70, Bruker, Karlsruhe, Germany) was used to measure the FTIR spectra of WF samples. The test conditions are as follows: 4,000–400 cm^–1^ scanning wave number range, 4 cm^–1^ resolution, and 64 s scanning time. All measurements were performed in triplicates. The OMNIC 8.2 software (Thermo Nicolet Inc., United States) was used to analyze all the measured data. FTIR spectra data in the range of 1,200–800 cm^–1^ were deconvolved and normalized. The short-range ordered structure of starch in WF was detected by calculating the absorbance ratio at 1,047/1,022 cm^–1^ (R_1047/1022_) (7, 9). The FTIR spectra were fitted and analyzed by the Peakfit software 4.12 (SPSS Inc., Chicago, IL, United States), and then the ratio of the secondary structure of the protein in WF was calculated based on the peak area. Amide I bands (1,600–1,700 cm^–1^) were assigned as follows: 1,612–1,618 cm^–1^, 1,625–1,635 cm^–1^, and 1,685–1,695 cm^–1^ were β-sheets; 1,640–1,655 cm^–1^ was random coils; 1,658–1,665 cm^–1^ was α-helixes; and 1,670–1,680 cm^–1^ was β-turns (10, 11).

### Determination of free sulfhydryl content

The determination of free sulfhydryl content of protein referred to the method of Suo et al. (12) with minor modifications. The samples were dissolved in 6 ml of Tris-Gly buffer (8 M Urea, 0.086 M Tris, 0.092 M Gly, 0.004 M EDTA, pH 8.0) and extracted with shaking for 60 min. After centrifugation for 20 min, the supernatant (4 ml) was taken out, and Ellman’s reagent (80 μl, 4 mg/ml DTNB in buffer) was added. The mixture of the two was reacted for 5 min, and then the absorbance was measured at 412 nm. The following formula was used to calculate the free sulfhydryl content of protein in WF:


$$C_{S\,H}=\frac{73.53\times A_{412}}{C}$$

*C* represents the concentration (mg/ml) of protein in the samples.

### Color analysis

Color measurements were conducted at room temperature with a Ci6x color difference analyzer (Aiselicai Technology Co., ltd., Shanghai, China). Before the testing, background calibration was performed using white and black. The measured brightness value (L*, 100 = white, 0 = black), red-green value (a*, positive value = red), and yellow-blue value (b*, positive value = yellow) were recorded (13). The following formula was used to calculate the whiteness index (WI) of WF.


$$W\,I=100-\sqrt{{\left(100-{\text{L}}^{*}\,\right)}^{2}+a^{*}\,{}^{2}+b^{*}\,{}^{2}}$$

The total color difference (ΔE) was calculated according to the following equation as a measure of the total color change between modified WF and WF (represented by an index 0).


$$△\,E=\sqrt{{\left(L^{*}-L_{0}^{*}\right)}^{2}+{\left(a^{*}-a_{0}^{*}\right)}^{2}+{\left(b^{*}-b_{0}^{*}\right)}^{2}}$$

### Solubility and swelling power

Samples were analyzed for solubility (S) and SP using the Chaple’s method (14). The sample (0.5 g, dry basis) and distilled water (25 ml) were added into a centrifuged tube and mixed well. The mixture was heated at 90°C for 30 min, taken out to cool, and centrifuged (3,000 r/min). After the separation of the supernatant and sediment, the supernatant was dried at 105°C to constant weight (W_1_). The weight of sediment in the centrifuge tube was recorded as W_2_. The weight of the sample was recorded as W. The S and SP were calculated by the following formula.


$$S\left(\%\right)=W_{1}/W\times 100$$

$$S\,P\,\left(g/g\right)=W_{2}/\left(W-W_{1}\right)$$

### Rapid viscosity analysis

The viscosity of WF was analyzed by a Rapid Viscosity Analyzer (RVA4500, Perten Instruments, Hägersten, Germany). The samples (2.5 g, dry basis) were added into an aluminum can, and then distilled water was added to make the total weight of 28 g. After mixing evenly, the mixture was put into the measuring tank of RVA for analysis. The test conditions were as follows: equilibrated at 50°C for 1 min, heated to 95°C at 12°C/min, kept warm for 2.5 min, cooled down to 50°C at the same rate and held for 2 min. The speed in the first 10 s was 960 r/min, and the rest was 160 r/min. Finally, the viscosity curve was obtained.

### Dynamic rheological analysis

The rheological properties of WF were tested by a rheometer (Discovery HR-1, TA instrument Inc., Newcastle, DE, United States). The samples were prepared according to the method of Solaesa et al. (15) and modified appropriately in specific operations. The linear viscoelastic region was determined using a shear strain at 25°C at a scanning frequency of 1 Hz. The dynamic frequency was 0.1–10 Hz, and the shear strain was 1%. The diameter of the test probe is 40 mm, and the gap is 1 mm. The prepared WF pastes were put on the flat mold, and the edge was smeared with methyl silicone oil to prevent the sample from volatilizing. The storage modulus (G’, Pa), loss modulus (G”, Pa), and loss tangent (tan δ) of WF were obtained.

### Statistical analysis

All experiments were repeated at least three times, and the data were obtained from the average and standard deviation (SD) of all repeated measured data. ANOVA and Duncan’s multiple comparison tests (*p* < 0.05) were performed using SPSS statistics 26.0 software (IBM, Chicago, IL, United States) to determine significant differences between means. Graphs were made using Origin 2018 (Origin-Lab Inc., Northampton, MA, United States).

## Results and discussion

### Physicochemical properties of plasma-activated water

The pH value, conductivity, and ORP of DW and PAW are shown in Table 1. The pH value represents the hydrogen ion concentration in an aqueous solution. Compared with DW (6.44), the pH value of PAW decreased significantly to 2.68 (*p* < 0.05) after plasma treatment for 2 min. This indicated that the plasma treatment of DW would lead to the acidification of water. This may be due to the decomposition of N_2_ and O_2_ by the plasma discharge to generate nitrogen oxides, which were decomposed into H^+^, NO_2_^–^, and NO_3_^–^ after the reaction with water, resulting in a decrease in pH value in PAW (16).

**TABLE 1:** Physicochemical properties of DW and PAW.

Water	pH	Conductivity (μ S/cm)	ORP (mV)
DW	6.44 ± 0.33^a^	3.57 ± 0.27^b^	284.67 ± 1.62^b^
PAW	2.68 ± 0.01^b^	832.58 ± 4.76^a^	581.50 ± 2.13^a^

Values are expressed as means ± standard deviation of three measurements. Means with different lowercase letters in the same column indicate significant differences (p < 0.05).

Conductivity can be used to measure the ability of a solution to conduct electricity. Plasma treatment significantly increased the conductivity from 3.57 (DW) to 832.58 μS/cm (PAW) (*p* < 0.05). This may be related to the pH of PAW and the formation of NO_3_^–^ and NO_2_^–^ after plasma treatment, resulting in an increase of conductivity (17).

ORP can represent the redox properties of the solution. After plasma treatment, ORP significantly increased from 284.67 (DW) to 581.50 mV (PAW) (*p* < 0.05). The increase of ORP indicated that various ROS and RON, such as H_2_O_2_, O_3_, OH, and ONOO^–^, were produced by the plasma treatment (18).

### Granular morphology

**FIGURE 1:**
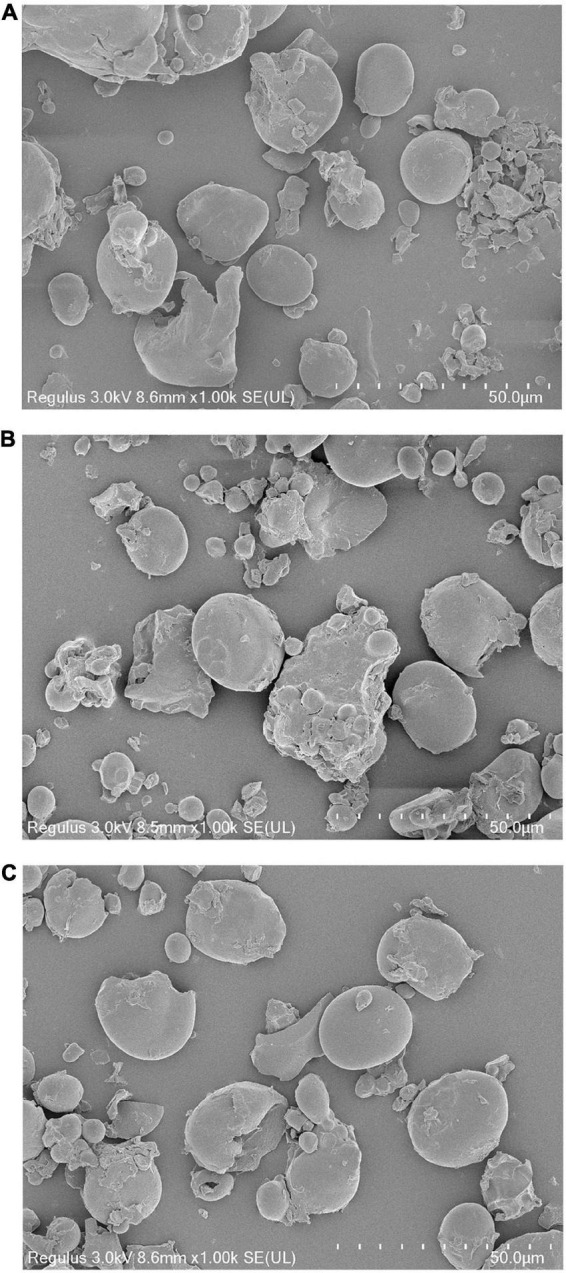
SEM images of WF and modified WF. **(A)** WF; **(B)** DW-WF; and **(C)** PAW-WF.

The scanning electron microscopy (SEM) observation results of WF and modified WF are shown in Figure 1. WF was mainly composed of starch particles, which were irregular, spherical, and rough on the surface. The rough part of the surface of starch particles and the debris around the particles are non-starch components, such as protein. After ANN treatment, a part of the starch particles was destroyed. This may be due to excessive water and heat during ANN, causing the gelatinization of part of the starch and, thereby, breaking the starch granules. In addition, the granular morphology of PAW-WF did not change significantly compared with DW-WF.

### Particle size distribution

**FIGURE 2:**
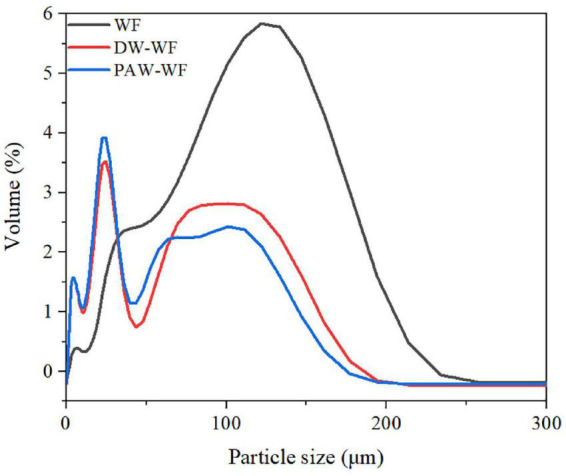
Particle size distribution of WF and modified WF.

The particle size distribution of WF and modified WF, and the size changes of the WF particles are shown in Figure 2 and Table 2. After ANN treatment, the large-sized particles in the WF were significantly reduced, while the smaller particles were relatively increased. This result may be explained by the fact that some starch granules were gelatinized and ruptured during ANN, which was in accordance with the observation results of SEM. Compared with WF, D (4.3), D10, D50, and D90 of DW-WF and PAW-WF were significantly lower (*p* < 0.05), which also indicated that ANN would lead to the destruction of starch granules instead of aggregation. This may be attributed to the cleavage of intermolecular hydrogen bonds in starch granules during ANN. Compared with DW-WF, the D (4.3), D10, D50, and D90 of PAW-WF were slightly lower. This trend may be due to the fact that during ANN, the acid component in PAW broke the molecular chain in starch, resulting in smaller and looser particles (6).

**TABLE 2:** Particle size, RC, short-range ordered structure of starch in WF, and modified WF.

Samples	D (4.3) (μ m)	D10 (μ m)	D50 (μ m)	D90 (μ m)	RC (%)	R_1047/1022_
WF	83.96 ± 0.12^a^	10.21 ± 0.39^a^	79.53 ± 0.64^a^	163.25 ± 1.39^a^	28.17 ± 0.15^a^	0.913 ± 0.019^a^
DW-WF	44.47 ± 1.17^b^	3.20 ± 0.00^b^	24.36 ± 0.23^b^	120.66 ± 4.74^b^	26.33 ± 0.15^b^	0.886 ± 0.030^a^
PAW-WF	38.45 ± 0.56^c^	3.15 ± 0.01^b^	23.43 ± 0.15^b^	105.02 ± 1.04^c^	25.30 ± 0.20^c^	0.878 ± 0.027^a^

Values are expressed as means ± SD of three measurements. Means with different lowercase letters in the same column indicate significant differences (p < 0.05). D (4.3) is the volume average diameter. D10, D50, and D90 are the particle sizes at 10, 50, and 90% of the volume of all particles, respectively.

### Crystalline structure

**FIGURE 3:**
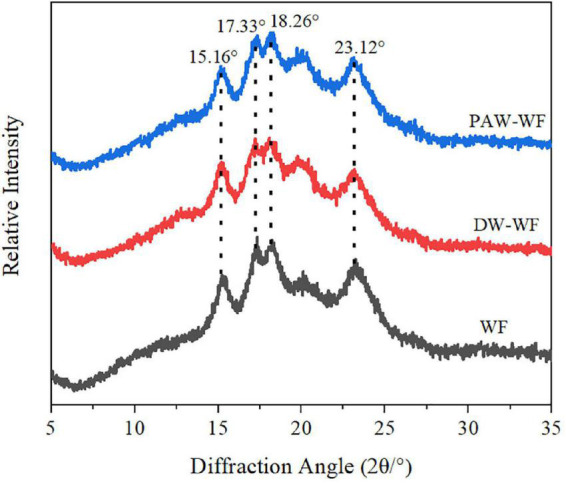
XRD patterns of WF and modified WF.

X-ray diffraction patterns and RC of WF and modified WF are shown in Figure 3 and Table 2. Both WF and modified WF showed obvious diffraction peaks at *2θ* = 15.16°, 17.33°, 18.26°, and 23.12°, which indicated that the crystal structure of starch in WF, DW-WF, and PAW-WF showed a typical A-type crystal structure (19). After ANN treatment, the position and peak shape of the diffraction peaks of WF did not change much, and no new characteristic peaks were generated. It can be concluded that ANN had no effect on the crystalline morphology of WF. However, ANN resulted in a significant decrease in the RC of the WF from 28.17 to 26.33% or 25.30% (*p* < 0.05). This is because the amylopectin structure in starch was destroyed, and the crystalline region was reduced during ANN. Moreover, partial starch granule gelatinization and double helical motion during processing may destroy starch crystallites or change crystallite orientation, which may also be the reason for the decreased RC (20). Additionally, PAW-WF had the lowest RC (25.30%) because the active species in PAW reacted with WF during PAW-ANN, resulting in the depolymerization of starch molecular chains (21). This was consistent with the findings of Zhu et al. (13), who studied the impact of plasma on the crystallinity of WF.

#### Short-range ordered structure of starch in wheat flour

**FIGURE 4:**
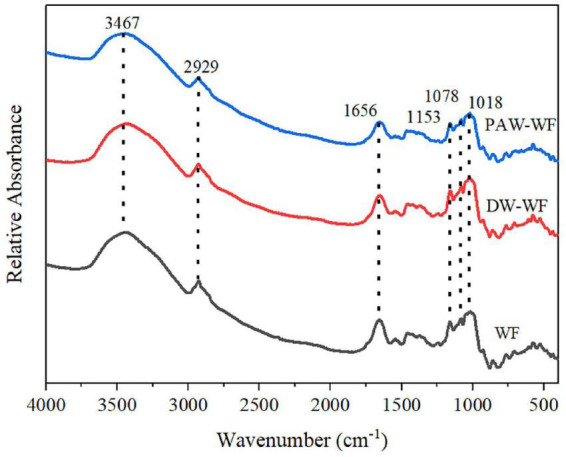
FTIR spectra of WF and modified WF.

As shown in Figure 4, the characteristic peaks of the FTIR spectra of WF and modified WF hardly changed. This indicated that the functional group of the modified WF was not affected. FTIR spectra in the range of 1,200–800 cm^–1^ are sensitive to short-range molecular order changes of starch. The absorption peaks of WF at 1,047 cm^–1^ and 1,022 cm^–1^ represent the ordered and disordered structures of starch molecules, respectively (22, 23). Therefore, R_1047/1022_ is used to identify the degree of the ordered structure of starch molecules. The larger R_1047/1022_, the higher the degree of short-range molecular order. According to Table 2, R_1047/1022_ did not significantly change (*p* < 0.05), indicating that the short-range ordered structure of starch in the modified MF was not changed.

#### Secondary structure and free sulfhydryl content of protein in wheat flour

FTIR spectroscopy is commonly used to characterize the secondary structure of the protein. There are several characteristic absorption bands in the FTIR spectroscopy of protein, among which the amide I band (1,600–1,700 cm^–1^) is often used to study their secondary structure. After PAW-ANN and DW-ANN, the secondary structure of the protein in WF was changed significantly (Table 3). Compared with WF and DW-WF, the content of β-sheets and β-turns were significantly reduced to 30.67 and 18.32% (*p* < 0.05), respectively, while the content of random coils and α-helices were significantly increased to 34.96 and 16.04% (*p* < 0.05), respectively, which indicated that the stability of the protein decreased, the hydrophobic groups inside the protein were exposed, and the partially ordered structure was transformed into a random coil structure after PAW-ANN. The increased irregular structures inhibited starch crystal arrangement, resulting in the decrease of RC, which was in consistent with XRD results.

**TABLE 3:** Secondary structure and free sulfhydryl content of protein in WF and modified WF.

Samples	β-sheets (%)	Random coils (%)	α-helices (%)	β-turns (%)	Free sulfhydryl (μ mol/g)
WF	36.72 ± 2.75^a^	25.20 ± 1.14^c^	12.67 ± 1.53^b^	25.41 ± 1.73^a^	10.68 ± 0.02^a^
DW-WF	36.18 ± 2.81^a^	28.86 ± 1.69^b^	13.39 ± 1.80^b^	21.57 ± 2.91^b^	5.72 ± 0.04^b^
PAW-WF	30.67 ± 1.36^b^	34.96 ± 1.49^a^	16.04 ± 1.42^a^	18.32 ± 1.44^c^	5.54 ± 0.02^c^

Values are expressed as means ± SD of three measurements. Means with different lowercase letters in the same column indicate significant differences (p < 0.05).

The free sulfhydryl content of WF and modified WF is shown in Table 3. After ANN treatment, the free sulfhydryl content of WF was significantly decreased (*p* < 0.05). This indicated the formation of disulfide bonds in proteins, which play an important role in protein aggregation. PAW-ANN further reduced the free sulfhydryl content from 10.68 to 5.54 μmol/g. This may be because ROS in the PAW led to the oxidation of free sulfhydryl in protein cysteine, aggravating the loss of free sulfhydryl (24).

#### Color

Whiteness is an important indicator of flour and its products. The white color of flour is required for many flour products (25). The color values and the total color difference between WF and modified WF are shown in Table 4. After DW-ANN, the L*, a*, and b* values of WF significantly decreased, while WI values significantly increased (*p* < 0.05). It was probable that ANN destroyed the flour structure and exposed the inner white core. Compared with DW-ANN, PAW-ANN decreased the L* and WI, while increasing a* and b*. This showed that the effect of PAW-ANN was slightly lower than that of DW-ANN. The larger the ΔE value, the greater the color difference. According to ΔE, the color between WF and modified WF was clear differences. Compared with DW-WF, the ΔE value of PAW-WF was larger. This might be due to the degradation and oxidation of starch caused by the acidic components and ROS in the PAW.

**TABLE 4:** Color, solubility, and swelling power of WF and modified WF.

Samples	L*	a*	b*	WI	ΔE	S (%)	SP (g/g)
WF	92.03 ± 0.02^a^	0.80 ± 0.01^b^	10.76 ± 0.04^a^	86.59 ± 0.04^b^	0	14.25 ± 0.12^b^	11.15 ± 0.14^a^
DW-WF	91.24 ± 0.01^b^	0.70 ± 0.02^c^	9.12 ± 0.07^c^	87.34 ± 0.04^a^	1.82 ± 0.09^b^	15.25 ± 0.27^b^	10.41 ± 0.49^a^
PAW-WF	90.51 ± 0.02^c^	0.85 ± 0.00^a^	9.27 ± 0.01^b^	86.71 ± 0.02^b^	2.13 ± 0.01^a^	18.75 ± 0.66^a^	10.52 ± 0.66^a^

Values are expressed as means ± standard deviation of three measurements. Means with different lowercase letters in the same column indicate significant differences (p < 0.05).

*Means to distinguish it from HunterLab.

#### Solubility and swelling power

According to Table 4, after ANN treatment, the SP of WF was not significantly changed, while the solubility significantly increased from 14.25 to 15.25% or 18.75% (*p* < 0.05). The increase in the solubility of DW-WF and PAW-WF may be because ANN destroyed the double helical structure of amylopectin in starch and enhanced the leaching ability of amylose (26). The solubility of WF treated with PAW-ANN (18.75%) was higher because of the degradation and oxidation of starch by the reactive species in the PAW (27), which was in consistent with XRD results. In addition, PAW-ANN could increase the solubility of protein due to the increase in irregular structures, which might also lead to increased solubility of WF.

#### Pasting properties

**FIGURE 5:**
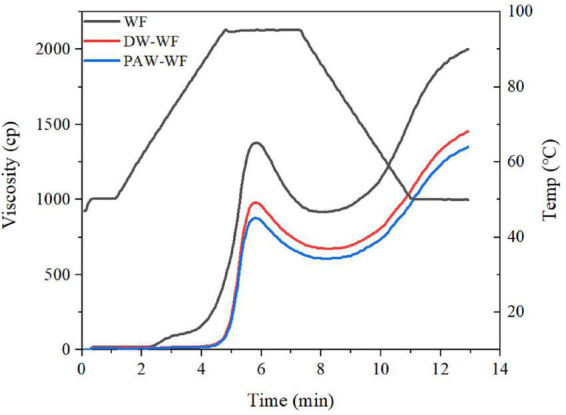
Pasting properties of WF and modified WF.

The viscosity properties of WF and modified WF are shown in Figure 5 and Table 5. ANN significantly reduced the peak viscosity, final viscosity, breakdown viscosity, and setback viscosity of WF (*p* < 0.05), which may be caused by the structural reorganization of starch granules. Similar findings were also demonstrated by Yadav et al. (28). Furthermore, the peak viscosity of PAW-WF was the lowest. It may be due to the hydrolysis of starch chains by the acidic components in the PAW, resulting in a decrease in the viscosity of WF (29). On the other hand, the acidic components in PAW also hydrolyzed the protein, resulting in a lack of protein network formation, which increases the brittleness of swollen starch granules, resulting in a decrease in viscosity of WF (30). The breakdown viscosity reflects the thermal stability of swollen starch granules at high temperatures and shear rates (20). Setback viscosity means that in the cooling stage of starch pastes, molecules can reassemble into ordered structures and form new crystals. The smaller the setback value, the more difficult it is for starch granules to form an extensive ordered structure when cooling (31, 32). Therefore, compared with WF and DW-WF, PAW-WF has better stability and weaker short-term retrogradation, suitable for quick-frozen flour products.

**TABLE 5:** Pasting properties of WF and modified WF.

Samples	Peak viscosity (cP)	Final viscosity (cP)	Breakdown viscosity (cP)	Setback viscosity (cP)
WF	1374.00 ± 7.07^a^	1997.50 ± 4.95^a^	463.00 ± 0.00^a^	1086.50 ± 2.12^a^
DW-WF	985.50 ± 4.95^b^	1462.50 ± 9.19^b^	310.50 ± 0.71^b^	787.50 ± 4.95^b^
PAW-WF	879.00 ± 1.41^c^	1355.50 ± 4.95^c^	274.00 ± 0.00^c^	750.50 ± 6.36^c^

Values are expressed as means ± SD of three measurements. Means with different lowercase letters in the same column indicate significant differences (p < 0.05).

#### Rheological properties

**FIGURE 6:**
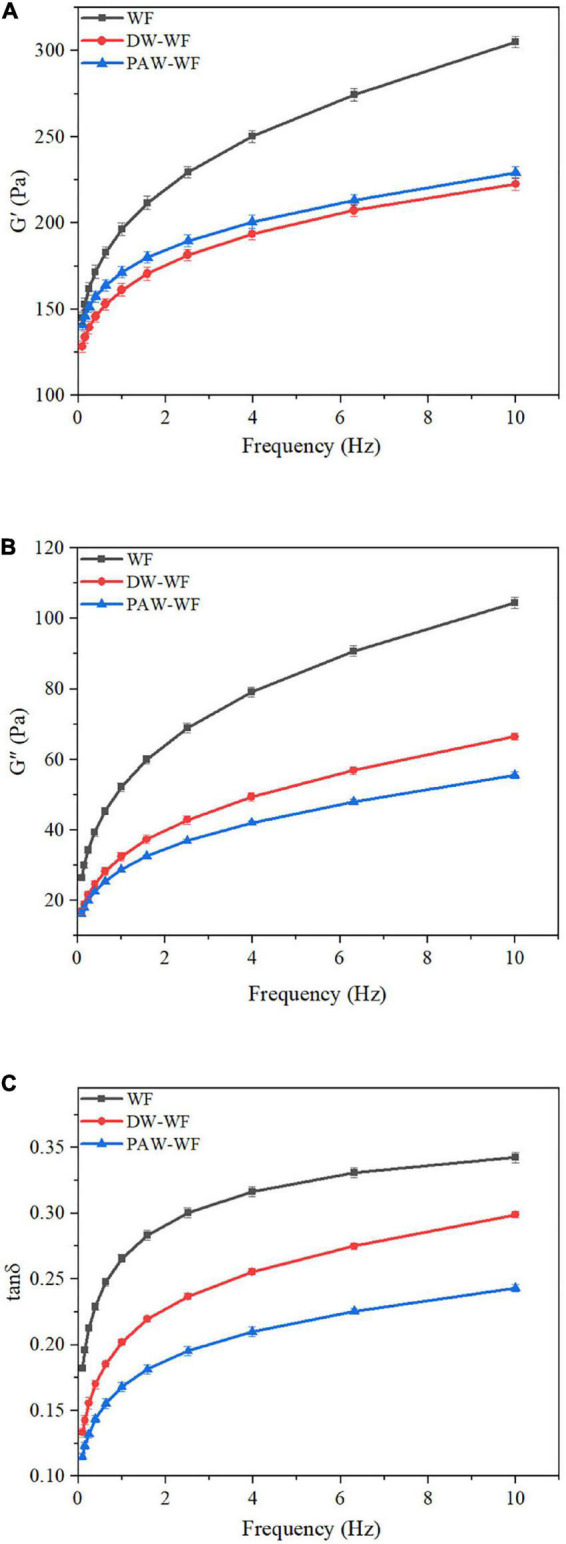
Dynamic rheological curves of WF and modified WF. **(A)** Storage modulus (G’) of WF, DW-WF and PAW-WF; **(B)** Loss modulus (G”) of WF, DW-WF and PAW-WF; and **(C)** Loss tangent (tanδ) of WF, DW-WF, and PAW-WF.

The dynamic rheological curves of WF and modified WF are shown in Figure 6. G’ and G” represent the elasticity and viscosity of gels, respectively (33). Tanδ represents the viscoelasticity ratio of the gel (34). According to Figures 6A,B, the G’ and G” values of both the WF and modified WF gels increased with the scanning frequency. G’ > G” (tanδ < 1) over the entire scanning frequency range indicated that all WF samples had the properties and elasticity of weak gels (35–37). ANN significantly reduced G’ and G” of native WF, indicating that ANN could significantly change the viscoelasticity of WF gels. Compared with WF, the gels of DW-WF and PAW-WF were softer and less viscous, consistent with the results of RVA. Similarly, after ANN, the tanδ values of WF gels decreased in the entire frequency range, indicating an increase in the proportion of elastic components in the WF. Moreover, compared with DW-WF, PAW-WF had lower tanδ and larger G’ value, indicating that PAW-WF had stronger gel properties with potential applications in the noodles. This might be due to more re-arrangement of amylose units formed on the surface of starch granules through larger pores and cracks. In addition, the formation of the amylose-protein complex on the surface of starch granules might also be another important reason.

## Conclusion

**FIGURE 7:**
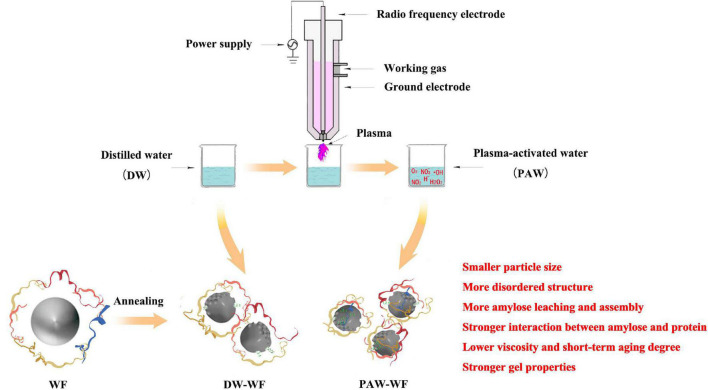
Influencing mechanism of PAW-ANN on the structure and properties of WF.

In summary, PAW-ANN had a significant influence on the structure and properties of WF due to the presence of acidic components and ROS in the PAW (Figure 7). Compared with DW-ANN, PAW-ANN decreased the particle size, long-range ordered structure of starch, and the partially ordered structure of the protein in WF, leading to more amylose leaching and assembly, and stronger interaction between amylose and protein. Furthermore, PAW-ANN increased the solubility, stability, and gel properties of WF, resulting in a lower peak viscosity and short-term aging degree of WF. Therefore, PAW-ANN would be employed as a novel physical modification technology to change the structure and improve properties of WF, especially for the “green” production of flour products requiring low viscosity, retrogradation rate, and high stability.

## Data availability statement

The original contributions presented in this study are included in the article/supplementary material, further inquiries can be directed to the corresponding author/s.

## Author contributions

YY contributed to the conception and design and wrote the first draft of the manuscript. YY and BN contributed to the funding of the study. XX, XuJ, ZC, and XiJ organized the database. YH and MS contributed to writing—review and editing. All authors contributed to the article and approved the submitted version.
